# Modeling and Machine Learning of Vibration Amplitude and Surface Roughness after Waterjet Cutting

**DOI:** 10.3390/ma16196474

**Published:** 2023-09-29

**Authors:** Michał Leleń, Katarzyna Biruk-Urban, Jerzy Józwik, Paweł Tomiło

**Affiliations:** 1 Faculty of Mechanical Engineering, Lublin University of Technology, ul. Nadbystrzycka 36, 20-618 Lublin, Poland; k.biruk-urban@pollub.pl (K.B.-U.); j.jozwik@pollub.pl (J.J.); 2 Faculty of Management, Lublin University of Technology, ul. Nadbystrzycka 36, 20-618 Lublin, Poland; p.tomilo@pollub.pl

**Keywords:** abrasive waterjet cutting, difficult-to-cut materials, vibration measurements, simulations, machine learning ML, material cutting

## Abstract

This study focused on analyzing vibrations during waterjet cutting with variable technological parameters (speed, *v_fi_*; and pressure, *p_i_*), using a three-axis accelerometer from SEQUOIA for three different materials: aluminum alloy, titanium alloy, and steel. Difficult-to-machine materials often require specialized tools and machinery for machining; however, waterjet cutting offers an alternative. Vibrations during this process can affect the quality of cutting edges and surfaces. Surface roughness was measured by contact methods after waterjet cutting. A machine learning (ML) model was developed using the obtained maximum acceleration values and surface roughness parameters (Ra, Rz, and RSm). In this study, five different models were adopted. Due to the characteristics of the data, five regression methods were selected: Random Forest Regressor, Linear Regression, Gradient Boosting Regressor, LGBM Regressor, and XGBRF Regressor. The maximum vibration amplitude reached the lowest acceleration value for aluminum alloy (not exceeding 5 m/s^2^), indicating its susceptibility to cutting while maintaining a high surface quality. However, significantly higher acceleration amplitudes (up to 60 m/s^2^) were registered for steel and titanium alloy in all process zones. The predicted roughness parameters were determined from the developed models using second-degree regression equations. The prediction of vibration parameters and surface quality estimators after waterjet cutting can be a useful tool that for allows for the selection of the optimal abrasive waterjet machining (AWJM) technological parameters.

## 1. Introduction

The constitution of machine part geometries has been evolving rapidly for many years with the emergence of new technologies. Waterjet machining (AWJ) of materials, which involves the use of high-pressure water along with the addition of an abrasive, has become a popular method for shaping machine parts in various industries around the world [1]. Waterjet cutting is a technology that uses a high-pressure waterjet mixed with abrasives to cut a variety of materials, including composites, glass, steel, and multilayer structures [2]. The flexibility of this technology has made it widely used in the aerospace, automotive, and construction industries [3]. 

The optimal selection of cutting parameters is essential to ensure the stability and reliability of the cutting process, as well as to achieve a high-quality cutting surface. However, achieving these optimal cutting parameters can be a technological challenge, often due to process instability and insufficient data needed to stabilize the process [4,5]. Consequently, there is a need to develop strategies for monitoring and controlling the material cutting process with the primary objective of enhancing the cutting process quality and, subsequently, attaining the best possible cutting surface quality [6,7]. 

Works by other authors concerned, among other things, the quality of the machined surface obtained using waterjet cutting (WJC) technology in relation to the influence of selected dynamic parameters (feed rate, abrasive flow rate, and pressure) on the quality of the machined surface for AISI 316L steel [8,9].

Cutting materials through waterjet processing offers several advantages compared to other cutting methods, such as laser cutting or plasma cutting [10]. These benefits include the ability to cut through materials with diverse properties, even those that are challenging to cut using traditional methods, and the creation of a small heat-affected zone, leading to reduced thermal deformation. This is particularly crucial for precision cutting applications [11]. Additionally, in many cases, especially for rough machining, waterjet cutting eliminates the need for additional operations such as milling, resulting in time and cost savings [12]. Consequently, waterjet cutting has gained popularity as a surface treatment technology, surpassing other cutting methods, such as laser cutting and plasma cutting [12,13]. Various researchers have employed both vibration and acoustic emission for the real-time monitoring of the AWJ (abrasive waterjet) process [14,15]. According to the literature [16], the first group of methods focuses on tracking the vibration signal parameters of the process itself and the working elements and their impact on the final product’s quality. The second group is concerned with diagnostics and condition monitoring of machine components based on the acoustic emission signal, aiming to identify the sources of the emission. As for the first category, most experimental studies involve the extraction of synthetic indicators, such as the root mean square (RMS) of the monitored signal, which has proven to be sensitive to the process and its parameters, as well as the condition of working components [17]. 

One of the disadvantages of the abrasive waterjet (AWJ) cutting process is its inherent instability, leading to vibrations that negatively impact the quality of the cutting edge and surface [18,19]. However, monitoring these vibrations during the cutting process presents an opportunity to enhance the stability and reliability of the process, consequently influencing the quality of the surface and edges after cutting [20,21]. Experimental studies have demonstrated that vibrations can be effectively identified using an accelerometer attached to the workpiece being measured [22]. This approach was also utilized in the study [23], and it can be employed in further experimental investigations on measuring vibrations during the waterjet abrasive jet cutting of various materials, including composites, glass, and steel [24,25].

The use of a noninvasive vibration sensor to monitor vibration during abrasive waterjet cutting offers several advantages. First and foremost, it provides a practical and nonintrusive solution for a vibration monitoring system, which is crucial for ensuring the stability and reliability of the cutting process [26]. Additionally, this approach enables real-time vibration monitoring, providing valuable feedback that can be utilized to adjust cutting parameters and improve cutting quality [27]. 

In the AWJM (abrasive waterjet machining) process, high-speed abrasive particles suspended in a waterjet impact the workpiece surface, leading to vibrations in the workpiece and generating acoustic signals [28]. Researchers such as Peržel et al. [29]. have analyzed vibrations occurring during the cutting of stainless steel. In their study, the variable parameter was the abrasive mass flow rate, set at 250 and 400 g·min^−1^ (with a constant feed rate). The study measured amplitudes and frequency spectra to establish the relationship between the input factors of the AWJM process and the emission of vibrations and acoustic signals. Tyč et al. [21] investigated the cutting process of hard-to-machine materials (RSt 37-2 steel) of various thicknesses during AWJ. The study involved using three piezoelectric accelerometers as the core of a vibration monitoring station. The tests revealed a strong correlation between the root mean square (RMS) value of the signal and the feed rate. An increase in feed rate caused a corresponding increase in the RMS value, depending on the direction of vibration measurement by the accelerometer. Krenický and Rimár [30], in their research, measured vibrations to analyze the technological parameters of AWJM cutting. They employed nozzle stabilization and a specially designed workpiece clamping system to reduce vibrations. Another study by Karminis-Obratanski et al. [31] aimed to determine whether vibration measures could be utilized to monitor the efficiency of the AWJM process. The study concluded that there was no direct relationship between process efficiency and vibration amplitude [32,33]. However, they observed an increase in average vibration amplitude with the depth and width of the cut.

Zagórski et al. [34], on the basis of a study on the effect of varying parameters of waterjet cutting of cast aluminum, concluded that the parameter that has a significant effect on the surface roughness after cutting is the feed speed, *v_f_*.

An analysis of the literature indicates that there is still a gap in the research on the relationship between the vibrations occurring during abrasive waterjet cutting and the cutting surface quality of various materials. Therefore, it is important to properly select the machining parameters to achieve the lowest possible vibration in order to achieve the desired edge and cutting surface quality of the materials under analysis.

Methods involving artificial intelligence, such as machine learning (ML) methods, are increasingly employed in various research areas. ML methods are commonly used in different predictive tasks due to their capacity to forecast nonlinear systems and the simplicity of their deployment, which has led to their growing adoption in addressing research challenges associated with, for example, predicting hot flow stress [35], high-temperature deformation of steel, chemical composition modeling, industrial electrical tomography, and electrical impedance tomography [36,37,38]. Additionally, modeling has been used in research on abrasive waterjet machining. A study by Ganovska et al. [7] investigated the influence of roughness parameters (Ra, Rq, and Rz), technological parameters (traverse speed and abrasive mass flow rate), and vibration on the AWJC process for stainless steel. The study also derived equations to predict surface roughness parameters and concluded that the surface topography was affected by the traverse speed of the cutting head. Similarly, Ficko [1] examined the effects of selected technological parameters (traverse speed, depth of cut, and abrasive mass flow rate) during the machining of stainless steel by AWJ on the surface roughness (Ra) of the material [39,40]. The results from this study were adopted to develop a predictive model for the Ra parameter, using an artificial neural network (a type of ML method).

## 2. Materials and Methods

This research was conducted using an Eckert WaterJet COMBO cutting machine, which features a modern CNC controller ECK 872. This controller is operated by Windows XP and is linked to a touch screen, facilitating seamless interaction between the machine and the operator and enabling efficient control of the cutting process. The machine is equipped with an Ethernet connection and a USB interface, thus simplifying the transfer of programmed programs and leading to saved time and work optimization.

**Figure 1:**
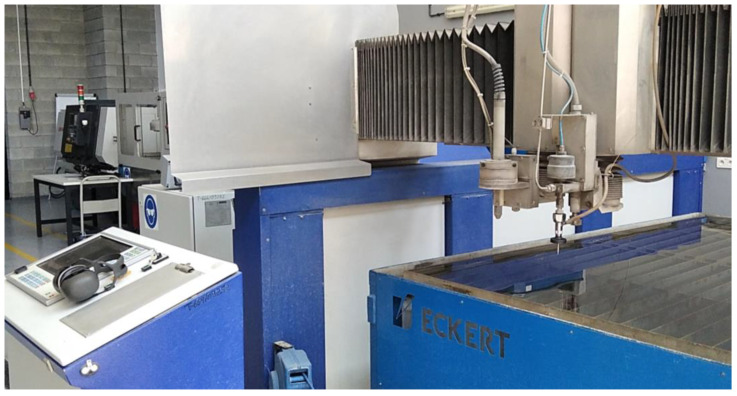
Eckert WaterJet COMBO portal cutting machine.

The Eckert WaterJet COMBO cutting machine (presented in Figure 1) is equipped with a high-pressure UHDE pump that is capable of generating a maximum pressure of up to 350 MPa. With this pump, the machine is capable of efficiently cutting various materials up to 150 mm thick, making it a versatile cutting tool. 

Samples made of titanium alloy, aluminum alloy, and steel were used in the study. Titanium Grade 5 (Ti-6Al-4V) is the most widely used titanium alloy, which is used in a wide range of industrial applications (from aerospace to medicine). It consists of 90% titanium, 6.4% aluminum, 4.1% vanadium, and other elements. Table 1 shows its chemical composition. 

Alloy 2024 is an aluminum–copper alloy that is widely used in the aerospace industry. It is known for its excellent strength and hardness, making it ideal for manufacturing parts that must withstand heavy loads, such as aircraft wings and fuselages. Table 2 shows the chemical composition of aluminum alloy 2024. 

S235JR is a grade of structural steel that is extensively used in various sectors of the economy, including the engineering industry and construction. This type of steel is favored as a structural material due to its widespread availability and excellent mechanical properties. Notably, S235JR steel is well regarded for its good weldability and formability, making it an ideal choice for structural applications. Additionally, its relatively soft nature makes it easy to cut and machine. For detailed information on the chemical composition of S235JR steel, please refer to Table 3.

The study used test specimens with dimensions of 200 × 80 × 15 mm. The height of the sample and, at the same time, the depth of the cut was 15 mm. In the course of the experiment, vibration measurements were carried out during the cutting of the samples with a water-abrasive jet, with varying process parameters. 

**Figure 2:**
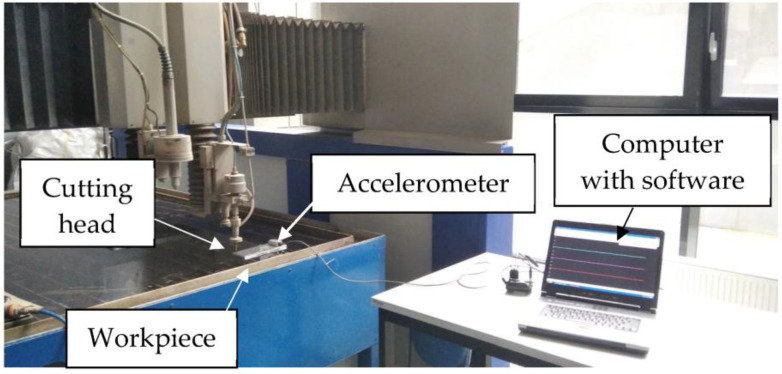
Test stand for vibration measurement.

The main component of the measurement station is shown in Figure 2.

**Figure 3:**
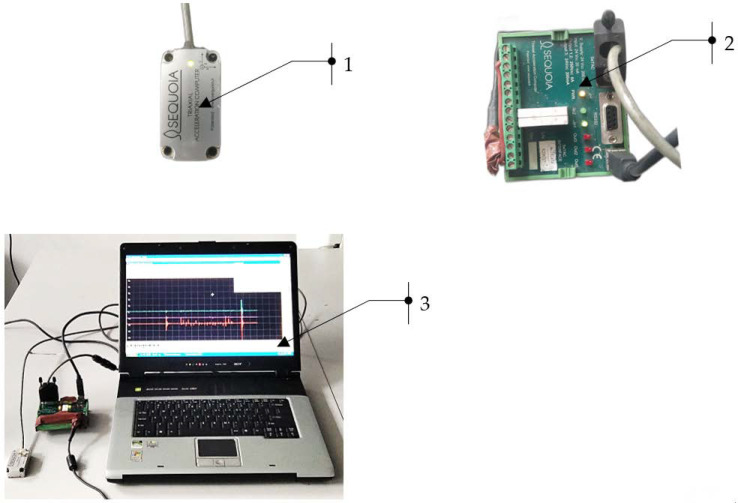
Measurement stand: 1—vibration sensor; 2—signal transducer; and 3—computer for signal analysis.

The SeTAC (Sequoia) system consists of a specialized sensor that measures acceleration, which is connected to a high-precision transducer of the collected signals. The computer used in this system is equipped with SeTAC software V5.14.0, which is used to process and display the measurement results, as well as analyze them. Technical data of the accelerometer: measuring of range ±18 g, dynamic range of 85 dB for 10 Hz, and resolution of 1 mg for 10 Hz. Pictures of the measurement station are shown in Figure 3.

The software used in the research facilitates real-time vibration monitoring to optimize the cutting process by minimizing vibrations. To ensure precise measurements, a vibration sensor was securely attached to the object, using beeswax. This ensured a stable connection between the sensor and the material, allowing for the accurate transmission of the measured vibrations during the entire measurement process. Consequently, the sensor remains firmly attached to the measuring point throughout the tests.

During all the conducted tests, the position of the sensor was rigorously maintained in the same place and at a constant distance from the edge of the material being cut. This consistent positioning is vital for obtaining high-quality data, enabling accurate and objective evaluation of the vibrations produced during cutting.

The primary objective of this research was to evaluate the influence of input technological parameters on vibrations during the cutting process of various materials. The key parameters analyzed during the study were the working pressure *p* (MPa) and the cutting speed *v_f_* (mm/min). These parameters are critical in waterjet cutting technology, as they significantly impact the cutting efficiency and quality [10]. This research focused on analyzing the effect of technological cutting conditions on the value of acceleration and their amplitude, *a* (m/s^2^).

A measurement of the 2D surface roughness was carried out after cutting the samples with variable technological parameters. The roughness measurements were performed on a Hommel tester T1000 contact profilometer (Villingen-Schwenningen, Germany). The measurements were carried out in five repetitions at one measuring point located 10 mm from the upper edge of the sample. In the tests, the following 2D surface roughness parameters were measured: Ra, Rz, and RSm.

**Figure 4:**
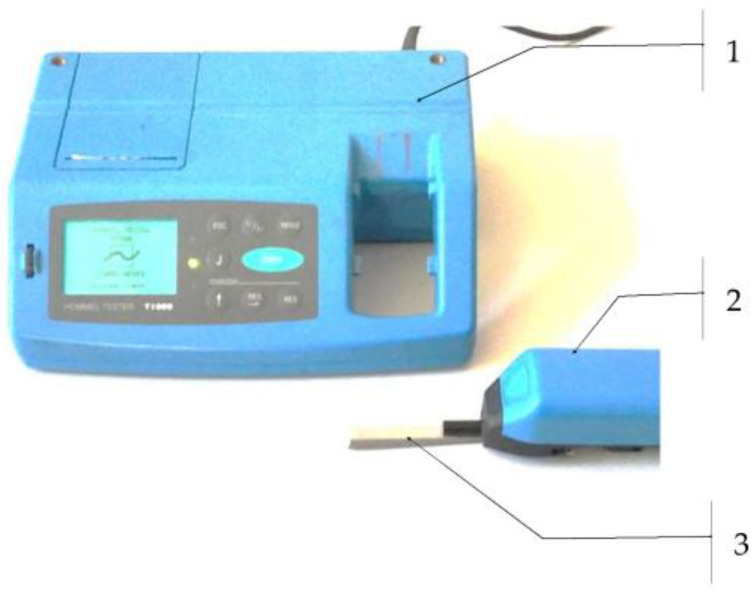
Surface roughness measurement by the Hommel tester T1000: 1—signal analysis device; 2—measuring head with needle; and 3—measured sample.

The surface roughness measurement using the Hommel tester T1000 is shown in Figure 4

**Figure 5:**
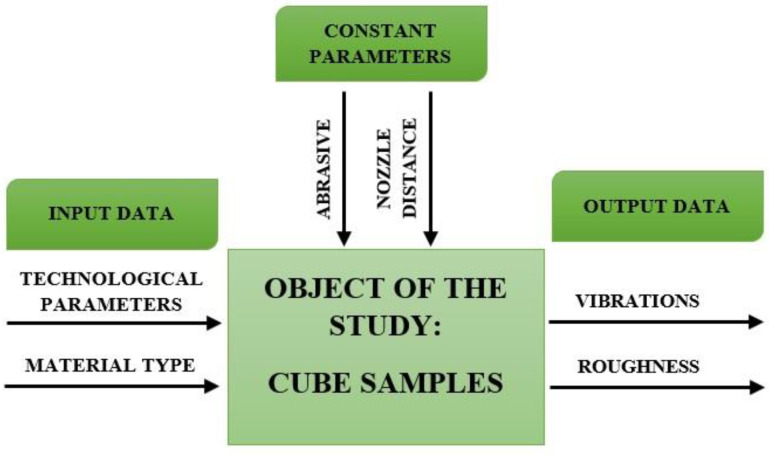
Research model.

In the experiment, as shown in Figure 5, the researchers employed a research plan with input variables that were modified to observe their effect on the cutting process. The process variables included the working pressure, *p_i_* (MPa) and the cutting speed, *v_fi_* (mm/min). These variables were systematically altered to study their impact on the cutting process and vibrations. 

Table 4 shows a summary of the constant technological parameters of cutting, i.e., the mass flow rate of abrasive *m_a_* (g/s), the distance of the nozzle from the material being cut *h* (mm), and the type of abrasive.

In order to thoroughly investigate the effect of varying process parameters (shown in Table 5), a series of experiments were conducted with different combinations of input parameter values. Four different working pressures (*p*_1_ = 350 MPa, *p*_2_ = 300 MPa, *p*_3_ = 250 MPa, and *p*_4_ = 200 MPa) and four different feed speeds (*v_f_*_1_ = 30 mm/min, *v_f_*_2_ = 40 mm/min, *v_f_*_3_ = 50 mm/min, and *v_f_*_4_ = 60 mm/min) were used. In addition, three construction materials were used: steel S235JR (*b*_1_), aluminum alloy Al2024 (*b*_2_), and titanium alloy Ti-6Al4V (*b*_3_).

## 3. Results and Discussion

**Figure 6:**
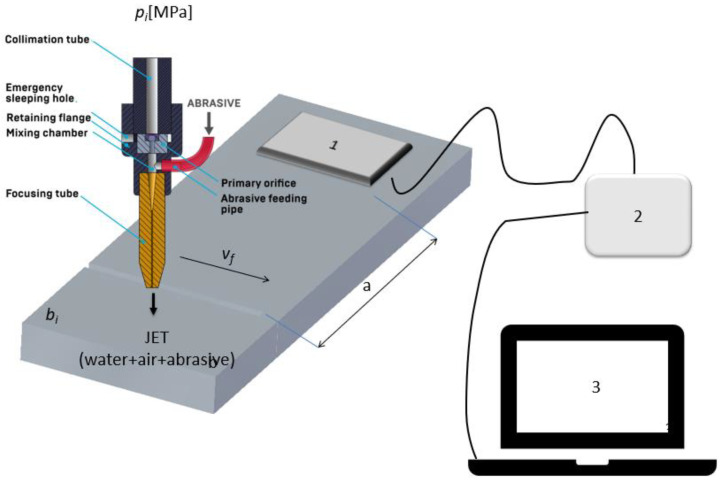
Schematic of the test stand: 1—vibration sensor; 2—signal transducer; and 3—computer for signal analysis with software.

Figure 6 presents a schematic of the experimental tests conducted, illustrating the key elements used in the process. The diagram depicts the nozzle utilized for cutting through the test material. The nozzle moved at a predetermined feed speed (*v_f_*) relative to the material being cut, represented as *b*. This controlled movement of the nozzle allowed for the precise management of the cutting parameters, avoiding the influence of uncontrolled fluctuations in the displacement rate. Vibrations generated during the cutting process were recorded by the vibration sensor, indicated as 1 in the diagram. The vibration signal transducer, marked as 2, converted the recorded sensor signal into a format interpretable by the computer system, designated as 3. This conversion allowed for data interpretation and enabled a rapid analysis to correct the technological parameters of the experiment. To ensure the stability and repeatability of the process and eliminate additional factors that could impact the test results, the cut sample was securely clamped to the table. This stable mounting of the sample was crucial for consistent and reproducible results. The vibration sensor was fixed at a fixed distance of *a* = 100 mm from the cutting points. This standardized distance ensured that the vibrations were consistently measured at the same location, enabling accurate comparisons and the reliable analysis of the data. 

**Figure 7:**
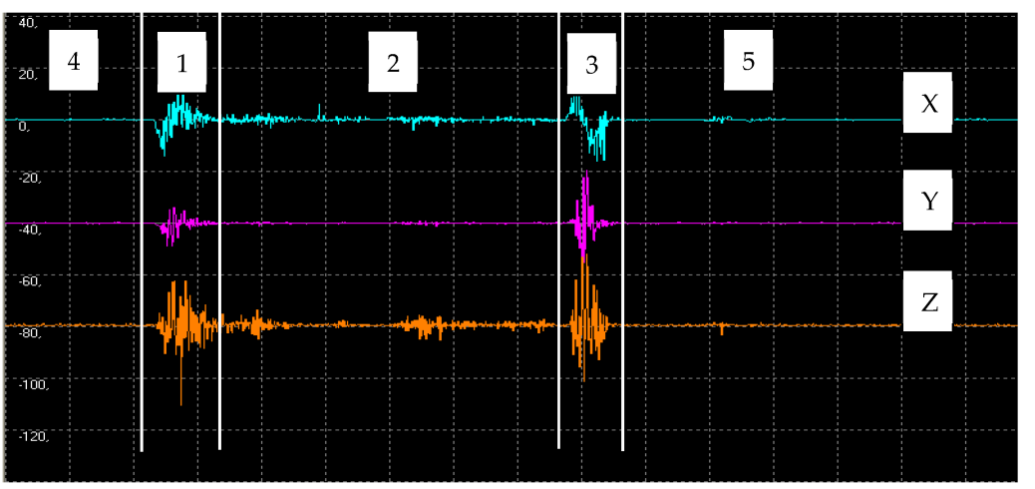
Time course of vibration with marked cutting areas: entry zone (1), stabilized cutting zone (2), and exit zone (3).

Figure 7 shows an example of vibration waveforms for steel (*v_f_* = 30 mm/min; *p* = 350 MPa). The presented waveforms marked with different colors correspond to the signals recorded on each axis: X, Y, and Z. From the example waveform, it can be seen that there are three main zones in the water-wall cutting process: the entry zone (1), the zone of stabilized cutting process (2), and the exit zone (3). In the entry zone, the increase in vibration is due to the impact of the water-abrasive jet on the surface of the water and coming into contact with the surfaces of the material being cut (zone marked 1). The effect of this, due to the strong force interaction, is an increase in the amplitude of vibration, the stabilization of which occurs only in the second zone. The second zone, marked No. 2 of the stabilized cutting process, in the full material, is characterized by a stabilized value of vibrations. During the exit of the jet from the cut material (zone three marked No. 3), an increase in vibrations is again noticeable, resulting from the exit of the jet from the material and the jet hitting the water surface again. The initial (4) and final (5) signal waveforms mark the period before the cutting process begins and ends.

This article presents only selected vibration time waveforms describing the studied phenomenon.

**Figure 8:**
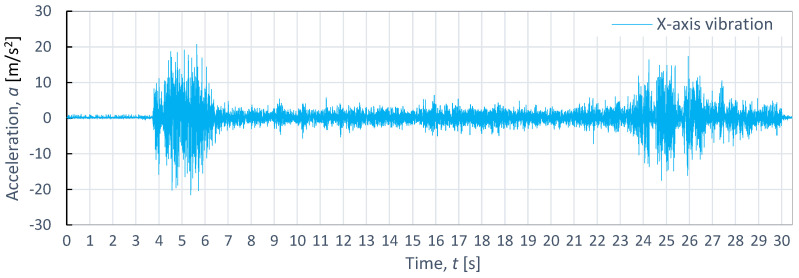
Example of time course of changes in acceleration *a*(*t*) (vibration) in the X-axis for aluminum alloy Al2024, as obtained at *p*_1_ = 350 MPa and *v*_2_ = 40 mm/min.

**Figure 9:**
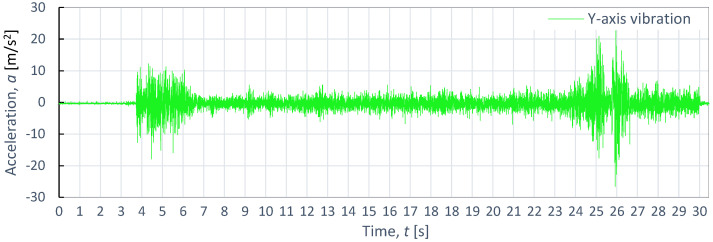
Example of time course of changes in acceleration, *a*(*t*) (vibration), on the Y-axis for aluminum alloy Al2024, as obtained at *p*_1_ = 350 MPa and *v*_2_ = 40 mm/min.

**Figure 10:**
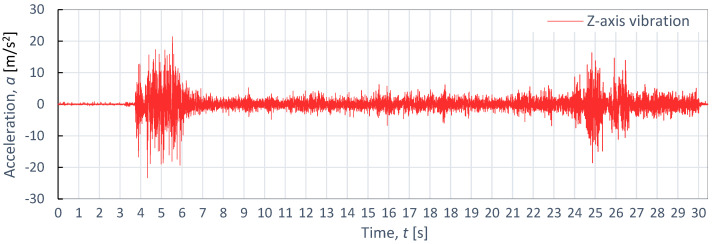
Example of time course of changes in acceleration, *a*(*t*) (vibration), on the Z-axis for aluminum alloy Al2024, as obtained at *p*_1_ = 350 MPa and *v*_2_ = 40 mm/min.

Figure 8, Figure 9 and Figure 10 showcases examples of vibration waveforms for the X-axis, Y-axis, and Z-axis recorded during the cutting process of aluminum alloy (Al2024) at specific technological parameters: pressure, *p*_1_ = 350 MPa; and speed, *v*_2_ = 40 mm/min. The presented time courses of vibration acceleration reveal three characteristic zones occurring during the cutting process. On the X-axis, the maximum vibration value of 20.7 m/s^2^ occurs in the entrance zone. On the Y-axis, the maximum vibration amplitude of 22.6 m/s^2^ is observed at the exit zone. Meanwhile, on the Z-axis, the highest vibration with an amplitude of 21.4 m/s^2^ occurs at the entrance zone. The increase in vibration amplitude at the exit zone can be attributed to the exit of the water-abrasive stream from the material and its impact on the surface of the water table. In the stabilized zone, which is characterized by comparable vibration values in all axes, the maximum value of vibration does not exceed 5 m/s^2^. This suggests that the cutting process stabilizes in this zone, leading to reduced vibrations and ensuring a higher quality of the cutting surface. 

**Figure 11:**
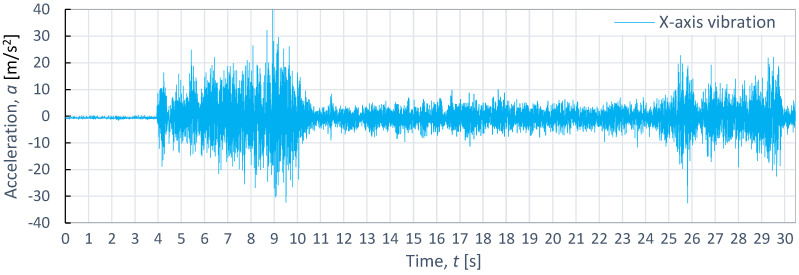
Example of time course of changes in acceleration, *a*(*t*) (vibration), on the X-axis for S235JR steel, obtained at *p*_1_ = 350 MPa and *v*_2_ = 40 mm/min.

**Figure 12:**
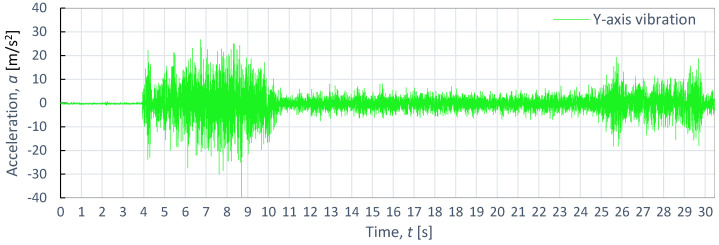
Example of time course of changes in acceleration, *a*(*t*) (vibration), on the Y-axis for S235JR steel, as obtained at *p*_1_ = 350 MPa and *v*_2_ = 40 mm/min.

**Figure 13:**
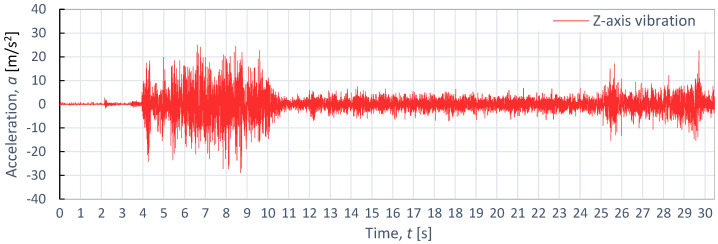
Example of time course of changes in acceleration, *a*(*t*) (vibration), on the Z-axis for S235JR steel, as obtained at *p*_1_ = 350 MPa and *v*_2_ = 40 mm/min.

Figure 11, Figure 12 and Figure 13 also display example vibration time waveforms on three axes (X, Y, and Z) for S225JR steel at process parameters of a pressure of *p*_1_ = 350 MPa and speed of *v*_2_ = 40 mm/min. Figure 11 presents the vibration signal for the X-axis, Figure 11 for the Y-axis, and Figure 12 for the Z-axis. An analysis of Figure 11, Figure 12 and Figure 13 reveals that, similar to the case of aluminum alloy (Al2024), three characteristic zones can be distinguished for S225JR steel: the entry zone (1), the stabilized cutting zone (2), and the exit zone (3). The entry zone (1) covers the period from the start of the cutting process until the waterjet comes into contact with the edges and surfaces of the material being cut. In this zone, significant changes in the waveform of the vibration signal are observed. Notably, this zone is longer for the S225JR steel being cut compared to the vibration time waveform for Al2024 aluminum alloy, despite maintaining the same technological cutting parameters. The zone of the stabilized cutting process refers to cutting through the full material, where, according to the study, no significant deviations in the values of the vibration amplitude were observed. This zone is shorter with respect to Al2024 aluminum alloy. The exit zone is characterized by an increased value of vibration amplitude. 

During the cutting of the S225JR steel, the entry zone was characterized by a longer duration compared to the exit zone observed during the cutting of aluminum alloy. Additionally, significant differences were noted in vibration values between the entry zone and the exit zone. In the exit zone, the vibration values were considerably smaller compared to those in the entry zone. In the entry zone, the highest vibration amplitudes were observed on the X-axis, reaching as high as 42.3 m/s^2^. For the Y-axis in the same area, the highest vibration amplitude was 39.3 m/s^2^, while for the Z-axis, the highest value of vibration amplitude was 28.8 m/s^2^. Conversely, in the stabilized zone, the vibration amplitudes were similar for all axes, not exceeding 10 m/s^2^. When compared to the results obtained for aluminum alloy, the percentage increase in vibration values for S225JR steel was 104.35% for the X-axis, 73.89% for the Y-axis, and 34.58% for the Z-axis, respectively.

The process of cutting steel involves the interaction of many factors, including material characteristics and technological cutting parameters.

**Figure 14:**
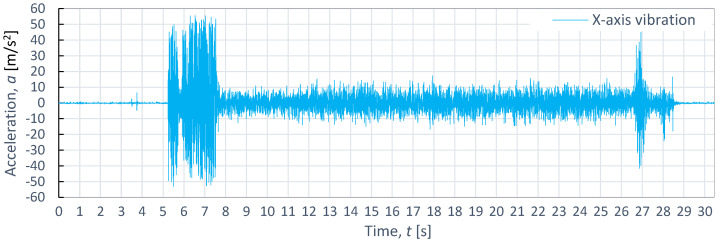
Example of time course of changes in acceleration, *a*(*t*) (vibration), on the X-axis for titanium alloy Ti-6Al4V, as obtained at *p*_1_ = 350 MPa and *v*_2_ = 40 mm/min.

**Figure 15:**
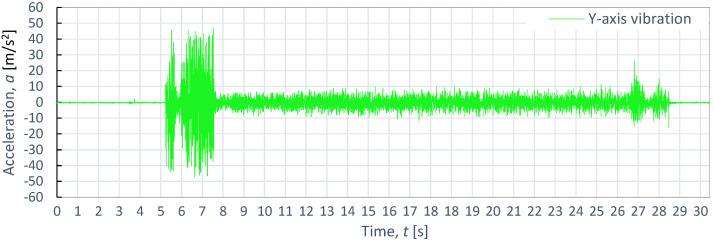
Example of time course of changes in acceleration, *a*(*t*) (vibration), on the Y-axis for titanium alloy Ti-6Al4V, as obtained at *p*_1_ = 350 MPa and *v*_2_ = 40 mm/min.

**Figure 16:**
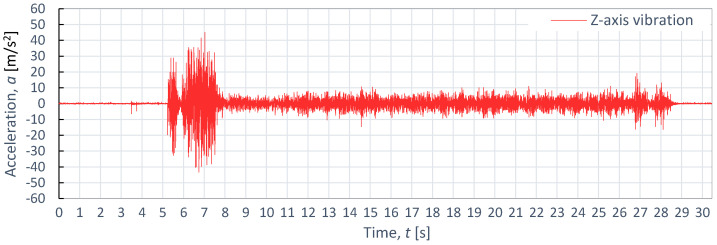
Example of time course of changes in acceleration, *a*(*t*) (vibration), on the Z-axis for titanium alloy Ti-6Al4V, as obtained at *p*_1_ = 350 MPa and *v*_2_ = 40 mm/min.

Figure 14, Figure 15 and Figure 16 show the results of the time course of vibrations during the cutting of titanium alloy Ti-6Al4V, as recorded during the cutting process with the same technological parameters as aluminum alloy and steel. The course of vibration characteristics during the cutting of titanium alloy Ti-6Al4V was characterized by the fact that, in the initial input zone for the X-axis, the vibrations were 167.45% larger with respect to the vibrations during the cutting of aluminum alloy and 31% larger compared to the vibrations recorded during the cutting process of steel. Their maximum amplitude value was 55.4 m/s^2^. 

Similarly, in the stabilized zone, compared to steel and aluminum alloy, the average vibration values were 5 m/s^2^ higher. The exit zone was shorter than in the case of steel.

**Figure 17:**
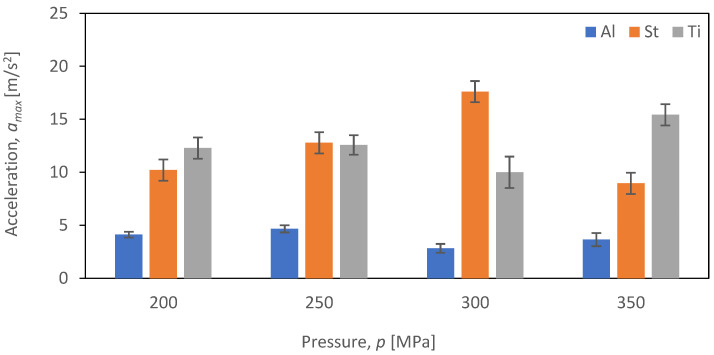
Summary of maximum *a_max_* values for a cutting speed of *v*_1_ = 30 mm/min at different pressures (*p_i_*) of different materials (*b_i_*).

**Figure 18:**
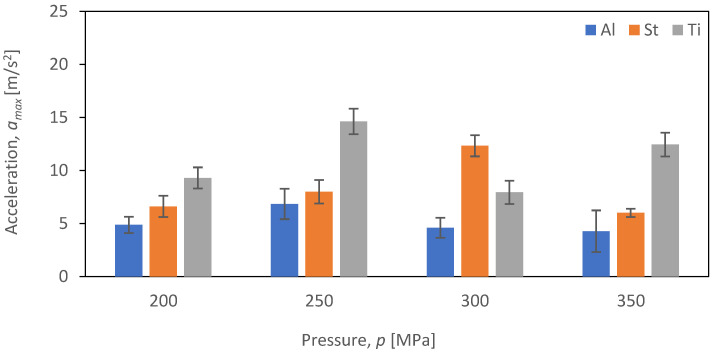
Summary of maximum *a_max_* vibration amplitude values for a velocity of *v*_2_ = 40 mm/min at different pressures (*p_i_*) for different materials (*b_i_*).

**Figure 19:**
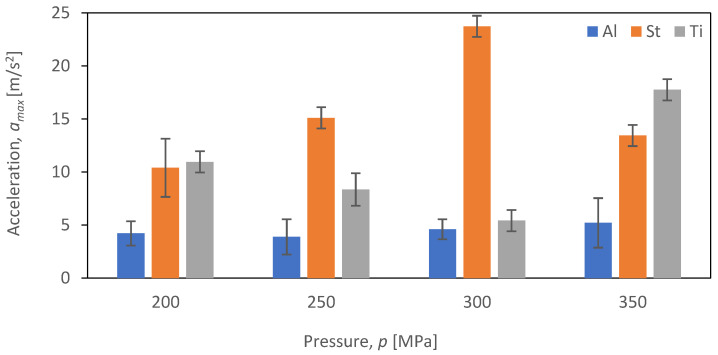
Summary of maximum *a_max_* for a velocity of *v*_3_ = 50 mm/min at different pressures (*p*_i_) for different materials (*b_i_*).

**Figure 20:**
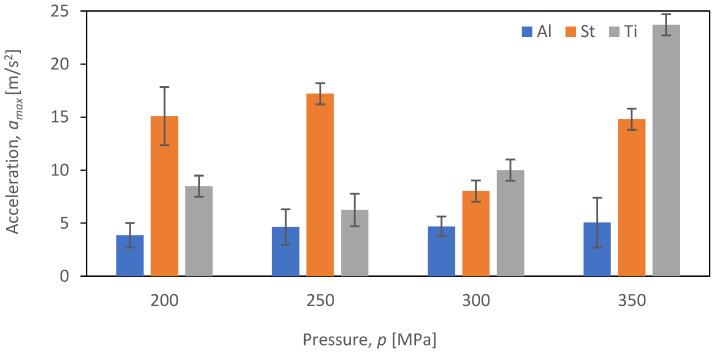
Summary of maximum *a_max_* values for a velocity of *v*_4_ = 60 mm/min at different pressures (*p_i_*) for different materials (*b_i_*).

The comparative evaluation of vibration time characteristics during the cutting of aluminum alloy Al2024, steel S235JR, and titanium alloy Ti-6Al4V was performed based on constructed graphs depicting the dependence of maximum acceleration (*a_max_*) as a function of pressure (*p_i_*) for four tested feed speeds (*v_i_*) for each of the selected materials subjected to the cutting process (*b_i_*). The analysis covered four different feed speeds: *v*_1_ = 30 mm/min (Figure 17), *v*_2_ = 40 mm/min (Figure 18), *v*_3_ = 50 mm/min (Figure 19), and *v*_4_ = 60 mm/min (Figure 20).

Figure 16 provides a comparison of the maximum vibration amplitude as a function of pressure (*p*) for speed (*v*_1_) and the three materials analyzed, namely aluminum alloy Al2024, steel S235JR, and titanium alloy Ti-6Al4V.

Figure 17 illustrates that the maximum value of vibration amplitude for aluminum alloy Al2024 remains consistently low (not exceeding 5 m/s^2^) regardless of the *p_i_* (pressure) value. Notably, Al2024 aluminum alloy has the lowest density among the three analyzed materials. On the other hand, for S235JR steel, there is a significant correlation between the *p_i_* (pressure) value and the maximum vibration values. An increase in pressure up to 300 MPa results in a proportional increase in vibration intensity when cutting S235JR steel. However, at a pressure of 350 MPa, there is a decrease in the maximum vibration amplitude for this material. In the case of titanium alloy, the highest values of maximum vibration amplitude were recorded at a pressure of 350 MPa, reaching 15.4 m/s^2^, while the lowest values were observed at a pressure of 300 MPa, measuring 10 m/s^2^. The decrease in vibration amplitude was 35.6%.

Figure 18 shows the dependence of the maximum values of vibration amplitude (*a_max_*) for the feed speed *v*_2_ = 40 mm/min.

Similar to the case of velocity *v*_1_, the maximum value of vibration amplitude reaches its lowest value for aluminum (not exceeding 6.8 m/s^2^) regardless of the pressure value (*p*). On the other hand, the highest value of maximum vibration amplitude was achieved when cutting titanium alloy Ti-6Al4V at a pressure of *p* = 250 MPa, measuring 14.6 m/s^2^, which is 114.7% higher than the maximum vibration amplitude obtained for aluminum alloy Al2024. A similar trend was observed when cutting S235JR steel—the values of maximum vibration amplitude increase with increasing pressure up to 300 MPa, after which, at the maximum pressure of 350 MPa, the maximum vibration amplitude decreases to 6 m/s^2^.

Figure 19 displays the values of maximum vibration amplitude for a feed speed of *v*_3_ = 50 mm/min.

For steel, a trend was observed for the maximum value of vibration amplitude to increase with the increasing pressure up to a value of 300 MPa. This increase assumes a maximum value at the same point among all analyzed velocities and reaches a maximum value of vibration amplitude of 23.7 m/s^2^ just at *v*_3_. At 350 MPa, there is a decrease in vibration. The lowest values of vibration amplitude were achieved, as in the case of other feed speeds, for aluminum alloy Al2024, not exceeding 5 m/s^2^. Analyzing the results for titanium alloy Ti-6Al4V, we can see that the lowest values of vibration amplitude were recorded at 300 MPa for each feed speed (*v*_1_ = 30 mm/min, *v*_2_ = 40 mm/min, *v*_3_ = 50 mm/min, and *v*_4_ = 60 mm/min). The lowest value of vibration amplitude was 17.7 m/s^2^.

In Figure 20, the maximum vibration values for *v*_4_ = 60 mm/min are presented.

At the highest feed speed, *v*_4_, the trend characteristic of lower feed speed for S235JR steel was not observed. Instead, there was no linear increase in the vibration amplitude values up to a pressure of 300 MPa, and the maximum vibration amplitude values for this material were reached at the lowest pressures of 200 and 250 MPa, measuring 15 m/s^2^ and 17.2 m/s^2^, respectively. The lowest value of maximum vibration amplitude for steel was 17.2 m/s^2^ at 250 MPa. Conversely, the highest value of maximum vibration amplitude of 23.7 m/s^2^ was achieved for titanium alloy Ti-6Al4V at the highest pressure, which was 282.26% higher than the value obtained for titanium alloy Ti-6Al4V at 250 MPa.

In the case of aluminum alloy Al2024, the value of maximum vibration significantly deviates from the values for the other materials regardless of the adopted feed speed, *vf*. This observed trend is consistent for all combinations of technological parameters, primarily due to the lack of a noticeable increase in vibration values when cutting Al2024 aluminum alloy.

These results highlight the importance of selecting the proper feed speed, *v_fi_*, to obtain optimal vibration amplitude results. A feed speed, *v_fi_*, that is too low can result in a slow and inefficient cutting process, while a feed speed that is too high can lead to an increased vibration. By finding the optimal feed speed, *v_fi_*, for each material (*b_i_*), it is possible to achieve a more efficient and high-quality cutting process. 

**Figure 21:**
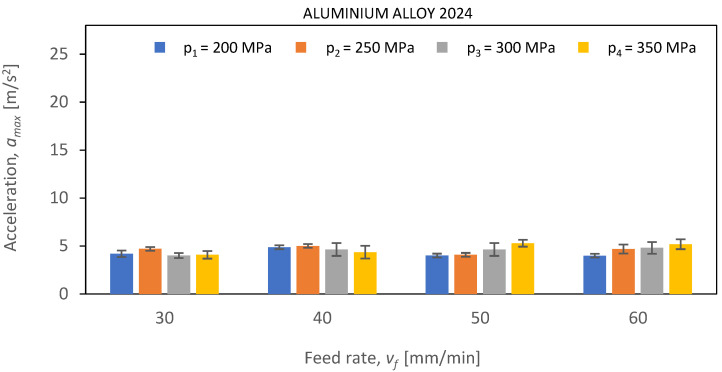
The course of changes in the amplitude value of *a_max_* for defined technological parameters of cutting: pressures of *p*_1_ = 200 MPa, *p*_2_ = 250 MPa, *p*_3_ = 300 MPa, and *p*_4_ = 350 MPa; and cutting speeds of *v*_1_ = 30 mm/min, *v*_2_ = 40 mm/min, *v*_3_ = 50 mm/min, and *v*_4_ = 60 mm/min for aluminum alloy Al2024.

**Figure 22:**
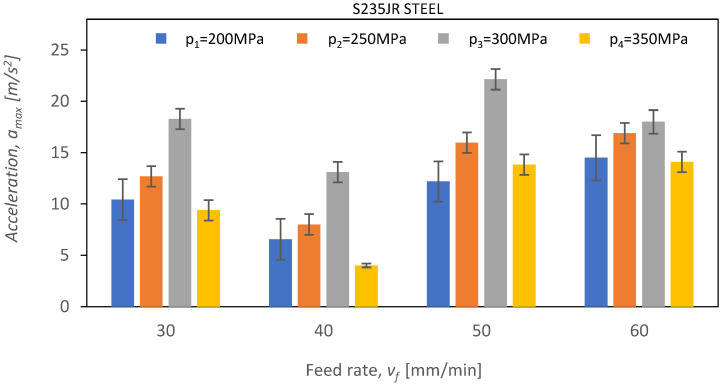
The course of changes in the amplitude value of *a_max_* for defined technological parameters of cutting: pressures of *p*_1_ = 200 MPa, *p*_2_ = 250 MPa, *p*_3_ = 300 MPa, and *p*_4_ = 350 MPa; and cutting speeds of *v*_1_ = 30 mm/min, *v*_2_ = 40 mm/min, *v*_3_ = 50 mm/min, and *v*_4_ = 60 mm/min for S235JR steel.

**Figure 23:**
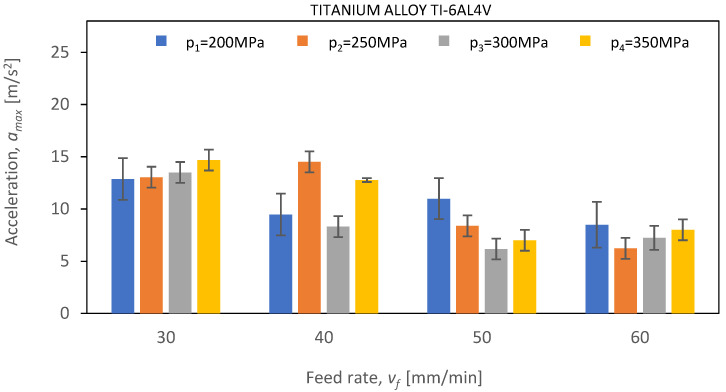
The course of changes in the amplitude value of *a_max_* for defined technological parameters of cutting: pressures of *p*_1_ = 200 MPa, *p*_2_ = 250 MPa, *p*_3_ = 300 MPa, and *p*_4_ = 350 MPa; and cutting speeds of *v*_1_ = 30 mm/min, *v*_2_ = 40 mm/min, *v*_3_ = 50 mm/min, and *v*_4_ = 60 mm/min for titanium alloy Ti-6Al4V.

Below, Figure 21, Figure 22 and Figure 23 show *a_max_* vibration amplitudes for three different materials: aluminum alloy, steel, and titanium alloy. By analyzing the vibration amplitudes and stabilization times in the X-, Y-, and Z-axis, one can better understand the physical basis of the cutting process under certain conditions. This information is very valuable for optimizing the cutting parameters and selecting the right materials for a given application.

Based on the analysis of the obtained experimental data and the determined estimates (Figure 21) during the cutting of Al2024 aluminum alloy, it is evident that the lowest values of vibration amplitude were recorded, regardless of the values of the technological parameters of cutting. The amplitude values of maximum vibrations (*a_max_*) do not exceed 5 m/s^2^. Throughout the adopted range of technological cutting parameters, the cutting process of the aluminum alloy remained stable. This stability can be attributed to several factors. Firstly, Al2024 aluminum alloy is characterized by the lowest density among the tested materials, allowing it to absorb a large amount of energy during the cutting process. Additionally, the alloy’s low hardness positively influences the machining process by minimizing the level of vibrations generated. These combined characteristics of Al2024 aluminum alloy contribute to the overall stability of the cutting process and result in low vibration amplitudes.

When analyzing the experimental data recorded during the cutting of steel (Figure 21), an increase in the value of vibration amplitude was observed compared to the cutting process of Al2024 aluminum alloy. Comparing the maximum vibration amplitude value for steel, which is 22.4 m/s^2^ at a pressure of *p*_3_ = 300 MPa and a feed speed of *v_f_* = 50 mm/min, with the same parameters for Al2024 aluminum alloy, a noticeable 380% increase in vibration amplitude is evident. Additionally, for the first three pressure values, namely *p*_1_ = 200 MPa, *p*_2_ = 250 MPa, and *p*_3_ = 300 MPa, a linear increase in vibration amplitude is noticeable. However, when the pressure changes to 350 MPa, there is a significant decrease in amplitude for all values of the feed speed, *v_f_*, amounting to as much as 75% at a feed speed of 60 mm/min. Steel is the material for which the waterjet cutting process implies the highest values of vibration amplitude. The highest value of vibration amplitude was achieved at a pressure of 300 MPa and a feed speed of *v_f_* = 50 mm/min, reaching 22.1 m/s^2^.

In the case of the water-abrasive cutting of titanium alloy Ti-6Al4V (Figure 22), a significant decrease in vibration amplitude values can be observed for feed speeds of *v_f_* = 50 mm/min and *v_f_* = 60 mm/min, amounting to 97.1% and 75% at 350 MPa with respect to the results obtained for S235JR steel. The highest values of vibration amplitude occur for the smallest value of feed speed, *v_fi_*, and do not exceed 15 m/s^2^. As the feed speed *v_fi_* increases, a decrease in vibration amplitude is observed. This trend is preserved for all values of pressure *p_i_*.

In conclusion, the analysis of peak acceleration values in the AWJM process provided valuable insights into the causal relationships between the technological conditions of the cutting process and the parameters describing the levels of mechanical vibrations generated during the hydro-abrasive cutting of various materials. This understanding of the phenomena occurring during hydro-abrasive machining can significantly impact the improvement of the efficiency and effectiveness of the AWJM process across a wide range of materials. Industries such as aerospace and biomedical, where hydro-abrasive machining is indispensable, stand to benefit greatly from these findings.

The obtained results offer valuable information for optimizing cutting conditions tailored to specific materials, while also aiding in the reduction of vibrations and their amplitudes to enhance the overall quality and efficiency of the cutting process. By implementing the knowledge gained from this research, it is possible to advance the capabilities of the AWJM process, making it a more reliable and efficient method for cutting various materials in critical industries.

**Figure 24:**
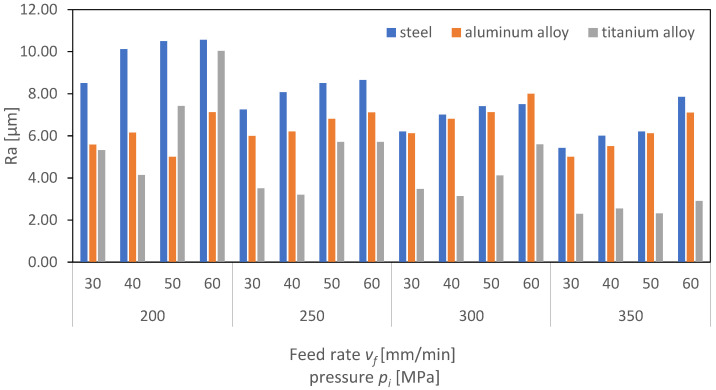
Dependence of parameter Ra on cutting pressure, *p_i_* (MPa), and feed rate, *v_f_* (mm/min), for the surface of aluminum alloy steel and titanium alloy.

**Figure 25:**
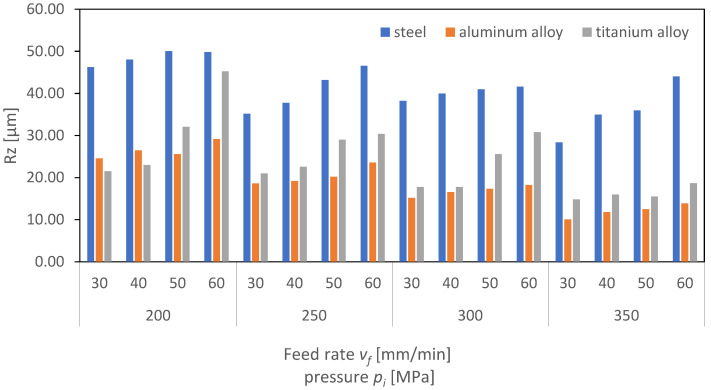
Dependence of parameter Rz on cutting pressure, *p_i_* (MPa), and feed rate, *v_f_* (mm/min), for the surface of aluminum alloy steel and titanium alloy.

**Figure 26:**
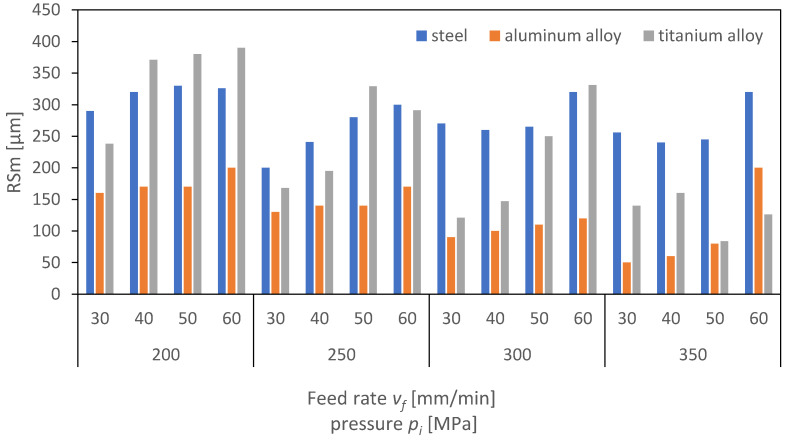
Dependence of parameter RSm on cutting pressure, *p_i_* (MPa), and feed rate, *v_f_* (mm/min), for the surface of aluminum alloy steel and titanium alloy.

Figure 24, Figure 25 and Figure 26 present the results of surface roughness for 2D parameters (Rz, Ra, and RSm) as a function of the feed rate and pressure values. Surface roughness parameters associated with the profile height are crucial for the proper interaction of surfaces of two machine elements. From the perspective of their interaction, it is advantageous for the surface roughness parameters to be as low as possible. As the charts indicate, the lowest roughness value for the Ra parameter is observed for titanium at higher pressures above 300 MPa. It can therefore be concluded that, for the analyzed case of aluminum and titanium, the obtained surface is characterized by a low friction coefficient.

Analyzing the charts, a trend can be identified indicating that, as the pressure increases, the roughness parameters decrease, but also, as the feed rate increases, the roughness increases. This trend is significant, as it suggests that to achieve a surface with minimal roughness and, hence, minimal friction, it is necessary to operate at higher pressures and lower feed rates. This is particularly important in industries where minimizing friction is crucial, such as in automotive or aerospace applications.

Moreover, it is interesting to note that aluminum, a material known for its strength and durability, exhibits lower roughness at higher pressures compared to titanium. This suggests that titanium might be a more suitable material for applications requiring lower surface roughness and, consequently, lower friction. However, the cost, availability, and machinability of titanium compared to aluminum must also be considered in the selection of materials for specific applications. The authors of the publication [31] found that, during the machining of titanium, they could not establish a direct correlation between the process outcome and the amplitude of vibrations. However, using a regression model, an increasing trend in the average vibration amplitude was observed as the depth and width of the cut increased from low values, through to medium values, and then to high values. This rise in the vibration amplitude is likely due to the higher kinetic energy and momentum of the abrasive waterjet in these cases.

Overall, the analysis of the charts provides valuable insights into the relationship between surface roughness, material type, pressure, and feed rate. These insights are essential for optimizing manufacturing processes and selecting the most appropriate materials for specific applications.

## 4. Numerical Modeling of Vibration and Roughness Parameters Using Machine Learning Models

Based on the obtained experimental studies, numerical modeling of vibration and roughness parameters was carried out using machine learning. Due to the characteristics of the data, five regression methods were selected:

- Random Forest Regressor;
- Linear Regression;
- Gradient Boosting Regressor;
- LGBM Regressor;
- XGBRF Regressor.

To choose a candidate for parameter optimization, the methods mentioned above were initially tested. The R^2^ (coefficient of determination) score, mean absolute error (MAE), and root mean square error (RMSE) metrics were used to compare the models’ levels of accuracy. 

**Figure 27:**
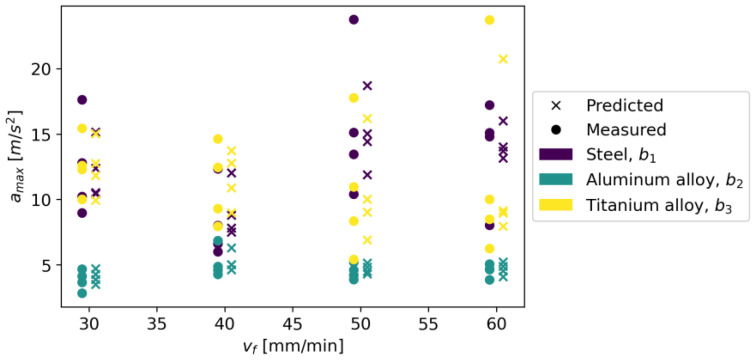
Measured and predicted values of acceleration (*a_max_*) vs. feed rate (*v_f_*) for Random Forest Regressor model (for steel, aluminum alloy, and titanium alloy).

**Figure 28:**
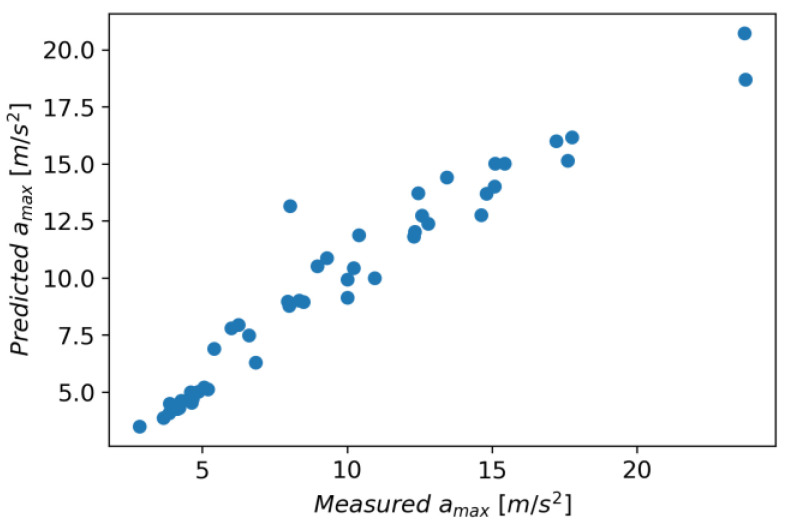
Correlation plot for measured and predicted values of acceleration, *a_max_* (for Random Forest Regressor model).

The initial parameters for the Random Forest Regressor were the loss function in the form of squared error and the number of trees in the forest equal to 100. It was assumed that the nodes expand until all leaves are pure or when the leaf contains less than two samples. Figure 27 shows measured vs. predicted values for Random Forest Regressor, and Figure 28 shows a correlation plot for measured and predicted values. This model achieved an R^2^ score of 0.920, an MAE equal to 0.990, and an RMSE of 1.460.

**Figure 29:**
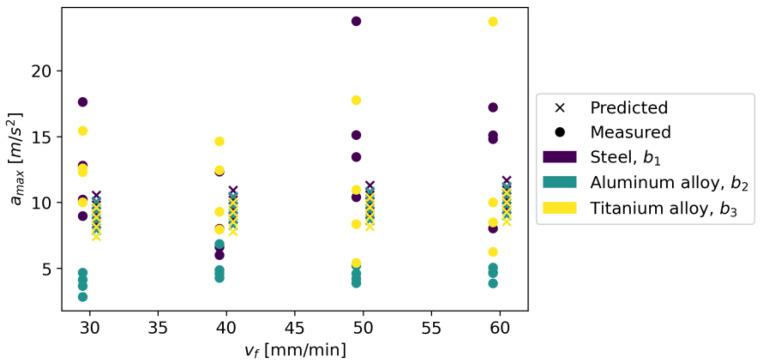
Measured and predicted values of acceleration (*a_max_*) vs. feed rate (*v_f_*) for Linear Regression model (for steel, aluminum alloy, and titanium alloy).

**Figure 30:**
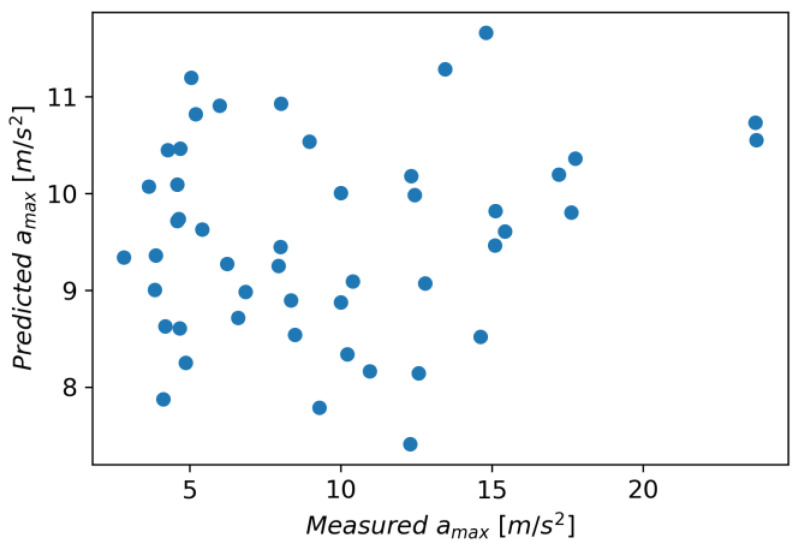
Correlation plot for measured and predicted values of acceleration, *a_max_* (for Linear Regression model).

The Linear Regression model achieved an R^2^ score of 0.037, an MAE of 4.280, and an RMSE of 5.070. Figure 29 shows measured vs. predicted values for the Linear Regression model, and Figure 30 shows correlation plot for measured and predicted values.

**Figure 31:**
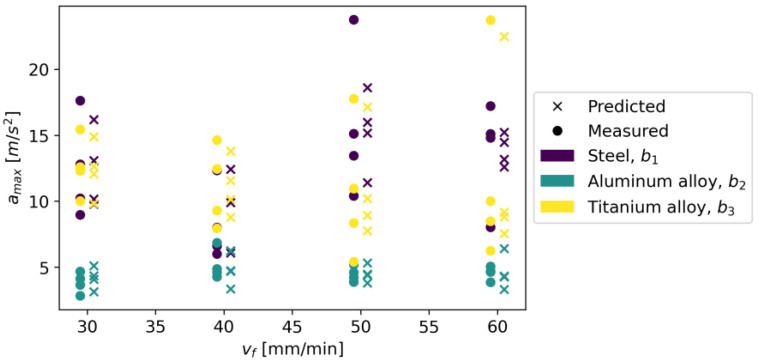
Measured and predicted values of acceleration (*a_max_*) vs. feed rate (*v_f_*) for Gradient Boosting Regression model (for steel, aluminum alloy, and titanium alloy).

**Figure 32:**
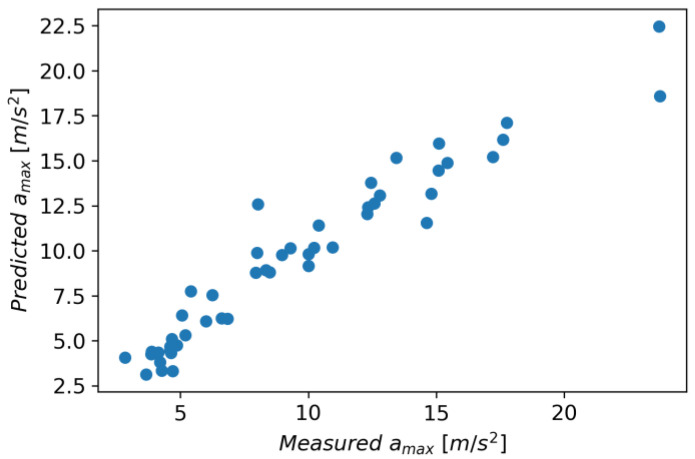
Correlation plot for measured and predicted values of acceleration, *a_max_* (for Gradient Boosting Regression model).

In Gradient Boosting, regressor models are built in an additive manner, which, at each stage, adjusts the regression tree to the negative gradient of a given loss function, which is assumed to be a squared error [41]. As other initial parameters, a learning rate of 0.1 and the number of boosting stages of 100 were also adopted. The model achieved an R^2^ score of 0.924, an MAE of 0.970, and an RMSE of 1.420. Figure 31 shows measured vs. predicted values for the Gradient Boosting Regressor, and Figure 32 shows a correlation plot for the measured and predicted values.

**Figure 33:**
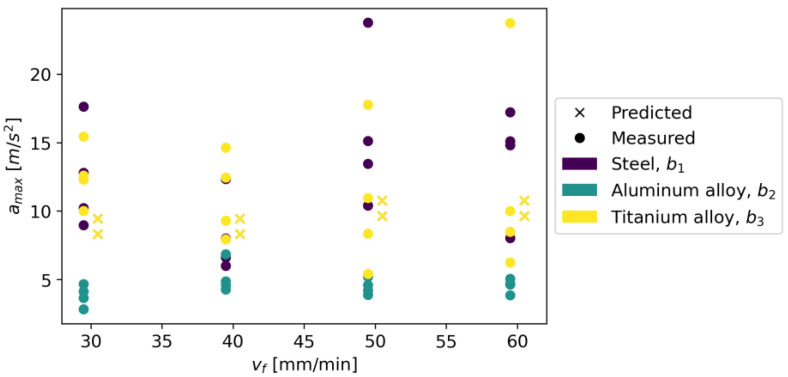
Measured and predicted values of acceleration (*a_max_*) vs. feed rate (*v_f_*) for LGBM Regressor regression model (for steel, aluminum alloy, and titanium alloy).

**Figure 34:**
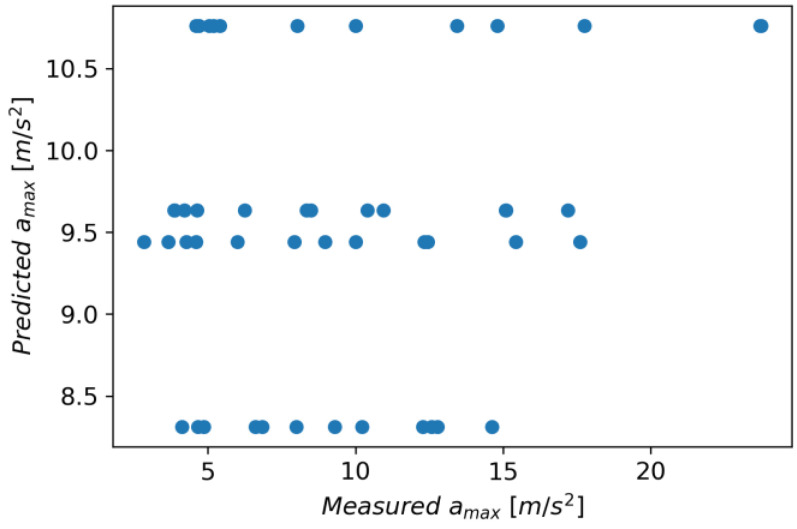
Correlation plot for measured and predicted values of acceleration, *a_max_* (for LGBM Regressor regression model).

In the LGBM Regressor model, the initial parameters were the number of leaves, 31; the learning rate, 0.1; and the number of boosted trees, 100. This model achieved an R^2^ score of 0.028, an MAE of 4.280, and an RMSE of 5.100. Figure 33 shows measured vs. predicted values for the LGBM Regressor, and Figure 34 shows correlation plot for the measured and predicted values.

**Figure 35:**
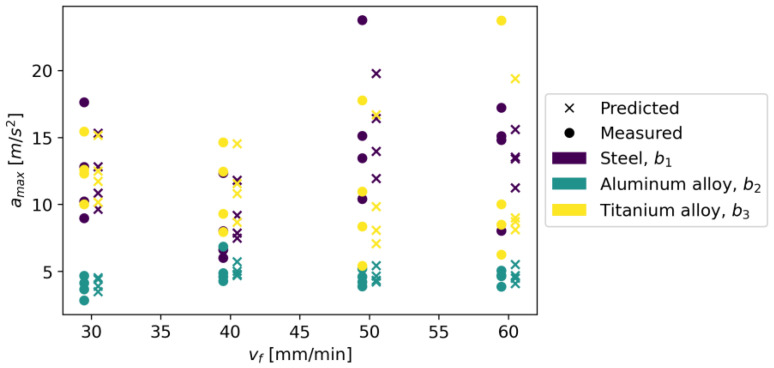
Measured and predicted values of acceleration (*a_max_*) vs. feed rate (*v_f_*) for XGBRF Regressor regression model (for steel, aluminum alloy, and titanium alloy).

**Figure 36:**
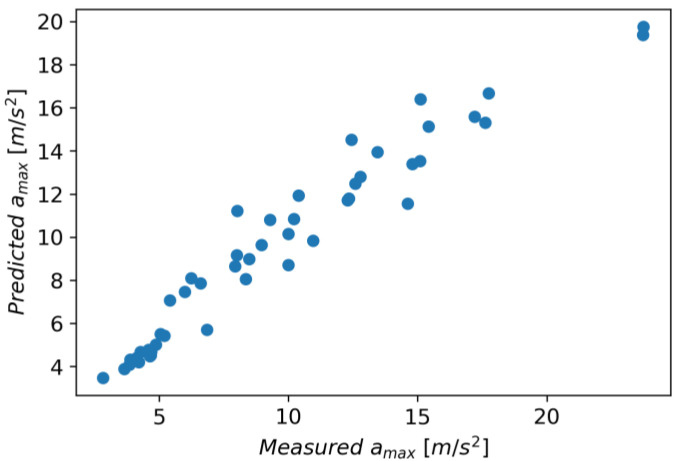
Correlation plot for measured and predicted values of acceleration, *a_max_* (for XGBRF Regressor regression model).

The following initial parameters were used for the XGBRF Regressor model: size of the forest equals 100, maximum depth of a tree is 6, and learning rate is 0.3. This model achieved an R^2^ score of 0.924, an MAE of 1.010, and an RMSE of 1.430. Figure 35 shows measured vs. predicted values for the XGBRF Regressor, and Figure 36 shows a correlation plot for the measured and predicted values.

**Figure 37:**
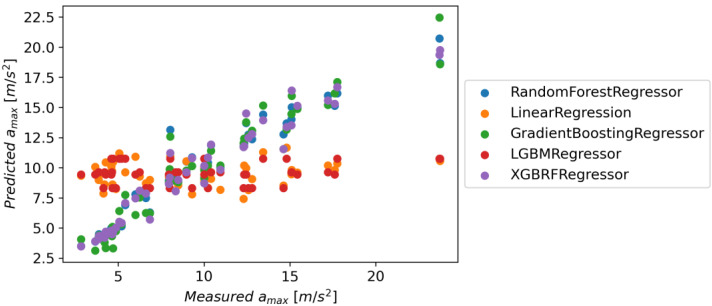
Predicted vs. measured values for selected models.

The metrics for individual models are presented in Table 6; additionally, Figure 37 shows the chart of actual vs. predicted.

The Gradient Boosting Regressor model has the highest accuracy for all metrics; the XGBRF Regressor model achieves the same value for the R^2^ score metric, but in the case of MAE and RMSE, its results are worse. The XGBRF Regressor and Linear Regression models achieved the weakest results among the selected models. Figure 37 shows the actual vs. predicted values for selected models.

The model with the best fit was selected for the optimization process—Gradient Boosting Regressor. The optimization process was carried out using the Grid Search algorithm with parameters defining the maximum depth of the individual regression estimators, $d_{max}$, and the number of boosting stages to perform, $e_{n}$. The $e_{n}$ parameter was optimized for the value from the set [110, 100, 90, 80, 60, 50, 45, 30, 20], and $d_{max}$ from the set [0, 2, 3, 4, 5, 6, 7, 8], where 0 means the expansion of nodes until all leaves are pure or the leaves contain less than two samples. After the optimization process, the following parameters were obtained: $d_{max}=0$ and $e_{n}=110$; the model obtained an R^2^ score that was increased by 0.0124, an MAE decreased by 0.7, and an RMSE decreased by 0.117 than the base model.

### Optimization of Process Parameters

Based on the developed Gradient Boosting Regressor model, the parameter optimization process was carried out using the differential evolution method [42]. The algorithm alters each candidate solution by combining it with other candidate solutions to produce a trial candidate at each run across the population. The best1bin strategy was adopted for optimization. Table 7 presents the optimal process parameters that minimize the vibration value.

**Figure 38:**
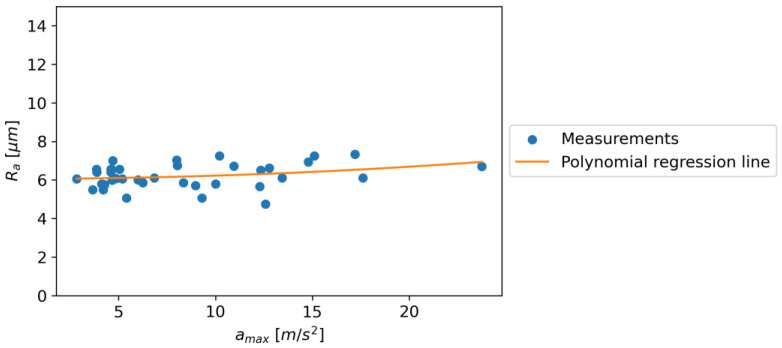
The dependence of Ra on *a_max_.*

In order to analyze the relationship between roughness and *a_max_*, a statistical analysis was carried out using polynomial regression. A second-order polynomial was used for the analysis because higher-order polynomials tended toward overfitting. In the case of the relationship between Ra and *a_max_*, a slight increase relationship was found. For the first-order polynomial adjustment, the slope of the linear function was 0.035. For the second-order polynomial fit, the obtained regression equation is Equation (1). Figure 38 shows the dependence of Ra on *a_max_*

Ra = 0.001∙k_2_∙*a_max_*^2^ + 0.002∙k_1_∙*a_max_* + 6.052∙k_0_ (1)

where k_0_, k_1_, and k_2_ are unit coefficients; and *a_max_* (m/s^2^) is the maximum acceleration.

**Figure 39:**
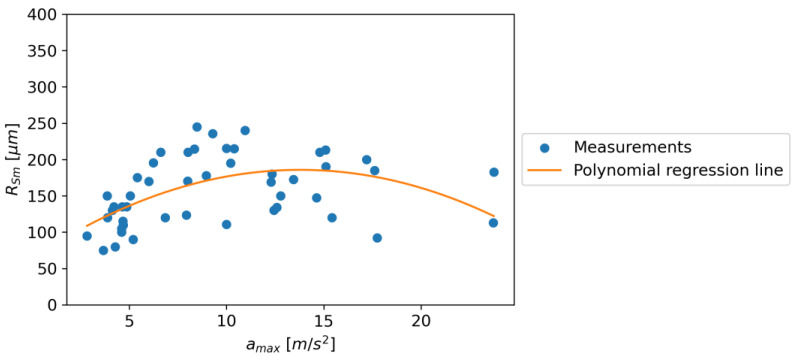
The dependence of RSm on *a_max_*.

In the case of the relationship between RSm and *a_max_*, for an increase in the value of *a_max_*, there is an increasing relationship to the value of 13.953, and then there is a downward trend. The following regression equation was obtained: Equation (2). Figure 39 shows the dependence of RSm on *a_max_*.

RSm = −0.642∙k_2_∙*a_max_*^2^ + 17.693∙k_1_∙*a_max_* + 63.811∙k_0_ (2)

where k_0_, k_1_, and k_2_ are unit coefficients; and *a_max_* (m/s^2^) is the maximum acceleration.

**Figure 40:**
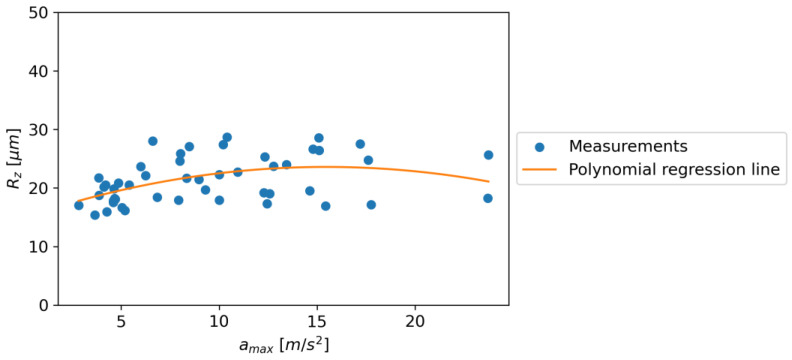
The dependence of Rz on *a_max_.*

The relationship between Rz and *a_max_* has similar characteristics to the relationship between Rz and *a_max_*, except that the relationship changes at an *a_max_* of 15.287. In the described case, the following regression equation was obtained: Equation (3). Figure 40 shows the dependence of Rz on *a_max_*.

Rz = −0.0363∙k_2_∙*a_max_^2^* + 1.123∙k_1_∙*a_max_* + 14.909 k_0_ (3)

where k_0_, k_1_, and k_2_ are unit coefficients; and *a_max_* (m/s^2^) is the maximum acceleration.

The coefficient of determination, R^2^, was Ra: 0.0804, RSm: 0.2871, and Rz: 0.2179, respectively. The results obtained from the polynomial regression analysis of the relationship between roughness parameters (Ra, RSm, and Rz) and *a_max_*, the maximum acceleration, reveal different trends. In the case of Ra and *a_max_*, a second-order polynomial regression model best fits the data, indicating a slight increase relationship with an Equation (1). This indicates that the roughness average (Ra) increases with the maximum acceleration (*a_max_*), although the relationship is not strong, as indicated by the small coefficients.

In contrast, the relationship between RSm and *a_max_* showed an increase until *a_max_* reached 13.953 m/s^2^, after which there was a downward trend, as indicated by Equation (2). This suggests that the mean peak-to-valley height (RSm) increases with *a_max_* up to a point, after which it starts to decrease. This could be due to the limitations in the material or equipment used in the experiment, which cannot sustain higher roughness beyond a certain acceleration.

Similarly, the relationship between Rz and *a_max_* showed a change at *a_max_* of 15.287 m/s^2^, as indicated by Equation (3). This suggests that the maximum height of the profile (Rz) increases with *a_max_* until 15.287 m/s^2^, after which it starts to decrease. This trend is similar to the one observed for RSm and could be due to the same limitations.

It is interesting to note that while Ra showed a continuous increase with *a_max_*, both RSm and Rz showed a peak and then a decline. This could be due to the nature of the parameters, where Ra is the arithmetic mean of the absolute values of the surface heights measured from a mean line and, hence, might not capture the peaks and valleys as accurately as RSm and Rz, which are based on the maximum and mean values of the peaks and valleys, respectively.

In conclusion, the relationship between roughness parameters and maximum acceleration is complex and varies for different parameters. While there is a continuous increase in Ra with *a_max_*, both RSm and Rz show a peak and then a decline at different values of *a_max_*. This could have implications in industrial applications where the roughness of a surface needs to be controlled or predicted based on the maximum acceleration used in the process. Further studies may be needed to understand the underlying mechanisms and to establish more accurate predictive models. 

## 5. Conclusions

This conducted experimental research focused on evaluating the influence of three key technological parameters of waterjet cutting, namely working pressure, *p_i_*; feed speed, *v_f_*; and material type (S235JR steel, Al2024 aluminum alloy, and Ti-6Al4V titanium alloy), on the amplitude of acceleration during the waterjet cutting process. 

This research aimed at identifying the optimal technological conditions for the cutting process in terms of surface quality and cutting efficiency.

Based on the research, the following conclusions were made:

1. The amplitude of vibration acceleration varies and depends on both the specifics of the material being cut and the controllable technological parameters of the cutting process.
2. Small changes in technological parameters such as the cutting speed (*v_fi_*) and pressure (*p_i_*) can lead to significant differences in the value of vibration acceleration amplitude.
3. During the analysis of the cutting process, three distinct and repeatable zones occurring during the cutting process were identified: the zone of entry of the water–waterjet into the material of the cut workpiece, the zone of stabilization of the process (during full cutting), and the zone of exit of the jet from the cut material.
4. Regardless of the adopted technological parameters of pressure (*p_i_*) and feed speed (*v_fi_*), the lowest values of acceleration were characterized by the process of cutting Al2024 aluminum alloy. The authors of the publication [34] state that, in the case of aluminum alloys, an increase in the stream velocity (*v_f_*) led to the deterioration of surface smoothness (resulting in higher roughness parameters). Modifying the height (h) of the sample did not have a consistent impact on the studied parameters. Although a strong correlation between the abrasive flow rate and the roughness characteristics of the sample surfaces was not observed, there was a slight tendency for the roughness of parts processed at higher abrasive flow rates to decrease.
5. Significantly higher values of vibration acceleration amplitude (reaching up to 60 m/s^2^) during cutting were registered for steel and titanium alloy for all zones and phases of the process (cutting zone, cutting zone, and exit zones).
6. A nonlinear effect of the pressure value (*p_i_*) and the feed motion speed (*v_fi_*) on the value of vibration amplitude during the cutting process was observed.

In the case of steel cutting, an increase in pressure up to the limit of 300 MPa causes a linear increase in maximum accelerations (Figure 21) at each of the analyzed feed speeds, *v_f_.* At the next pressure value of 350 MPa, there is a decrease in vibrations.

The results of the experiments presented in this study clearly show that vibration affects the efficiency of material removal during hydro-abrasive machining. In particular, it was noted that an increase in the amplitude of vibration leads to an increase in the rate of material removal. This observation is consistent with the results of studies by other authors, who noted that vibration can increase material removal rates by “breaking” the water layer between the abrasive particles and the machined surface, which facilitates the transport of abrasive particles into the machining zone.

However, our research also showed that there is a certain threshold of vibration amplitude beyond which the material removal rate begins to decrease. A possible explanation for this observation could be the variations in experimental parameters, such as the water pressure, abrasive particle size, and hardness of the material being machined.

In addition, our results suggest that vibration can affect the shape and quality of the surface after machining. In experiments with a higher vibration amplitude, post-treatment surfaces were rougher and showed a greater tendency to form microcracks. This phenomenon may be due to the higher interaction forces between the abrasive particles and the machined surface at increased vibration amplitudes.

Among the selected models (Random Forest Regressor, Linear Regression, Gradient Boosting Regressor, LGBM Regressor, and XGBRF Regressor), the Gradient Boosting Regressor model had the best fit to the data. This model achieved the highest values for the R^2^ score, MSE, and RMSE metrics, which were, respectively, 0.924, 1.010, and 1.430. The XGBRF Regressor model was also characterized by a high level of fit, as its R^2^ score was at the same level as the Gradient Boosting Regressor model, but the other metrics were worse. The Linear Regression and LGBM Regressor models were characterized by the smallest fit. The differential evolution method made it possible to determine the optimal process parameters that minimize the $a_{max}$ value.

The conducted studies provide valuable cognitive conclusions, allowing for the assessment of cause-and-effect relationships between the amplitude of acceleration in the waterjet cutting process and the technological parameters of cutting. This information is indirectly beneficial for optimizing the process and can significantly contribute to improving the efficiency and quality of cutting. As a result, it can lead to significant time savings in various cutting applications. By understanding how different technological parameters impact the vibration amplitudes, engineers and manufacturers can make informed decisions to enhance the overall performance and productivity of the waterjet cutting process.

## Figures and Tables

**Table 1:** Chemical composition of titanium alloy Ti-6Al4V (% mas.).

Al	V	C	Fe	O_2_	N_2_	H_2_	Ti
6.4	4.1	0.1	0.16	0.18	0.01	0.03	Rest

**Table 2:** The chemical composition of Al2024 alloy (% mas.).

Al	Cu	Mg	Mn	Fe	Si	Zn	Ti	Cr
93.5	3.8–4.9	1.2–1.8	0.3–0.9	≥0.5	≥0.5	≥0.25	≥0.15	≥0.1

**Table 3:** Chemical composition of S235JR steel (% mas.).

C	Mn	Cu	Al	Mo	Si	P	S	Fe
0.16	0.4	0.03	0.04	0.03	0.016	0.05	0.017	Rest

**Table 4:** Summary of constant technological parameters.

Technological Parameters	
Abrasive material	Garnet 80 mesh
Nozzle length	100 mm
Mass flow rate	8 g/s
Distance from the material being cut	3 mm

**Table 5:** Summary of the variable technological parameters.

No.	Material	Pressure *p_i_* (MPa)	Feed Speed *v_fi_* (mm/min)
1	Steel, *b*_1_; aluminum alloy, *b*_2_; titanium alloy, *b*_3_	350	30
2			40
3			50
4			60
5		300	30
6			40
7			50
8			60
9		250	30
10			40
11			50
12			60
13		200	30
14			40
15			50
16			60

**Table 6:** Models’ metrics.

Model	R^2^ Score	MAE	RMSE
Random Forest Regressor	0.920	0.990	1.460
Linear Regression	0.037	4.280	5.070
Gradient Boosting Regressor	0.924	0.970	1.420
LGBM Regressor	0.028	4.280	5.100
XGBRF Regressor	0.924	1.010	1.430

**Table 7:** Optimal parameters.

Material	Pressure $p_{i}$ (MPa)	Feed Rate $v_{fi}$ (mm/min)	$\mathrm{Estimated}\;a_{max}$
$\mathrm{Steel},\;b_{1}$	217.714	35.745	7.353
$\mathrm{Aluminum}\;\mathrm{alloy},\;b_{2}$	320.742	30.746	3.220
$\mathrm{Titanium}\;\mathrm{alloy},\;b_{3}$	279.784	49.452	6.855

## Data Availability

The data presented in this study are available upon request from the corresponding author.
